# Supplementary material for: Circadian clock mechanism driving mammalian photoperiodism
Source: Nat Commun. 2020 Aug 27;11:4291. doi: 10.1038/s41467-020-18061-z (PMC7453030; doi:10.1038/s41467-020-18061-z)
Supplement: Supplementary file 3 — Description of Additional Supplementary Information [file 41467_2020_18061_MOESM3_ESM.pdf]

Description of Additional Supplementary Files

File Name: Supplementary Data 1
Description: Seasonal epigenetic changes, MACs peak calling ChIP-seq H3k4me3

File Name: Supplementary Data 2
Description: Seasonal epigenetic changes, SICER differential peak analysis for H3K4me3

File Name: Supplementary Data 3.
Description: Seasonal gene expression, pairwise contrasts for RNA-seq

File Name: Supplementary Data 4
Description: CAGE transcription start site clusters and their relative seasonal abundance

File Name: Supplementary Data 5
Description: RNA-seq expression for known H3K4me3 modulators Supplementary Data 6: Diurnal gene expression, statistical analysis of 24 hour profiles from RNA-seq
